# Poly(amidoamine)-alginate hydrogels: directing the behavior of mesenchymal stem cells with charged hydrogel surfaces

**DOI:** 10.1007/s10856-018-6113-x

**Published:** 2018-06-30

**Authors:** André Schulz, Alisa Katsen-Globa, Esther J. Huber, Sabine C. Mueller, Asger Kreiner, Norbert Pütz, Michael M. Gepp, Benjamin Fischer, Frank Stracke, Hagen von Briesen, Julia C. Neubauer, Heiko Zimmermann

**Affiliations:** 1 Fraunhofer Institute for Biomedical Engineering, Joseph-von-Fraunhofer-Weg 1, 66280 Sulzbach, Germany; 2 Faculty of Medicine, Saarland University, Kirrberger Straße 100, 66421 Homburg, Germany; 3 Chair for Molecular and Cellular Biotechnology, Saarland University, 66123 Saarbruecken, Germany; 4 Faculty of Marine Science, Universidad Católica del Norte, Coquimbo, Chile

## Abstract

The surface charge of a biomaterial represents a promising tool to direct cellular behavior, which is crucial for therapeutic approaches in regenerative medicine. To expand the understanding of how the material surface charge affects protein adsorption and mesenchymal stem cell behavior, differently charged surfaces with zeta potentials spanning from −25 mV to +15 mV were fabricated by the conjugation of poly(amidoamine) to alginate-based hydrogels. We showed that the increase of the biomaterials surface charge resulted in enhanced quantities of biologically available, surface-attached proteins. Since different surface charges were equalized after protein adsorption, mesenchymal stem cells interacted rather with diverse protein compositions instead of different surface features. Besides an enhanced cell attachment to increasingly positively charged surfaces, the cell spreading area and the expression of adhesion-related genes integrin α5 and tensin 1 were found to be increased after adhesion. Moreover, first results indicate a potential impact of the surface charge on mesenchymal stem cell differentiation towards bone and fat cells. The improved understanding of surface charge-related cell behavior has significant impact on the design of biomedical devices and artificial organs.

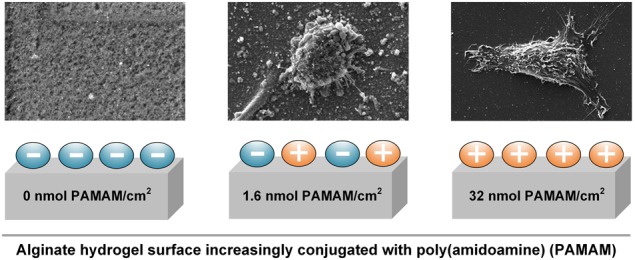

## Introduction

In the last years, interfaces between biomaterials and cells gained increasingly in importance for a wide range of medical and pharmaceutical applications (reviewed in [1, 2]). Since the success of a biomaterial is determined by its interaction with a biological system, the substrate is required to be both, biocompatible and functional. In principle, cells do not interact with the surface alone but rather with surface-attached proteins through direct binding to receptors within the cellular membrane [3]. Besides surface characteristics such as wettability [4], topography [5] and chemistry [6], the material surface charge is associated as a crucial parameter for cell-matrix interactions [7–15]. Here, same charges repel and opposite charges attract each other. Since proteins and cell membranes are net negatively charged due to the presence of phospholipids, proteins and polysaccharide conjugates [16], appropriate surface charges for cell-matrix contacts are net positive.

To date, mainly two concepts are described regarding the relationship of surface charge and cellular behavior: (I) An increased surface charge promotes cell attachment [11–14] and (II) positively charged surfaces induce differentiation processes of stem cells [7–10]. Existing studies mainly used acrylate-based substrates [9–13] or binary self-assembled monolayer systems deposited on a metal substrate [14]. So far, the researchers focused on the cell attachment of cell types such as fibroblasts [11, 14] and osteoblasts [12, 13] while neglecting to clarify the cell spreading in terms of cell area and morphology. Moreover, only the impact of the surface charge on the differentiation of stem cells towards bone tissue was examined with scientific effort [7–10] without considering other differentiation pathways of mesenchymal stem cells (MSCs), *e.g*. towards cartilage, fat or muscle cells. Therefore, the objective of the current work is to establish a hydrogel-based platform to expand the insight of how surface charges influence cellular behavior in respect to adhesion and differentiation.

On that account, we prepared specific surface charges adjusted by the conjugation of charged poly(amidoamine) (PAMAM) to alginate-based hydrogels. Alginate as a polysaccharide extracted from the cell wall of marine seaweed is one of the most applied biomaterials in biomedical sciences [17–20]. With its biocompatibility, its ability to form a hydrogel under mild conditions as well as adjustable mechanical and porous characteristics, alginate asserts itself as a widely applied biomaterial in tissue engineering [19–21] and drug delivery [22]. Since the alginate surface is negatively charged, protein adsorption was found to be low resulting in an inhibition of cell adhesion favorable for immunoisolations [23]. To address cell-matrix contacts, functional groups of the alginate structure can be modified in a versatile manner [20, 24]. In this study, we used highly positively charged PAMAM dendrimers, which are mainly applied as vehicles in drug delivery [25] and gene delivery [26]. With increasing generation PAMAM dendrimers possess a rising number of amino groups in the peripheral regions, which interact with surrounding molecules or cells. To study the impact of the surface charge on the cellular behavior, positive surface potentials can be fabricated by the conjugation of PAMAM to negatively charged alginate scaffolds. Here, MSCs are of great interest, since they have the potential to differentiate into mesodermal cell types (bone, cartilage, fat and muscle tissue) [27] and showed clinical importance in treating diseases with cell-based therapy [28].

With the current work we provide poly(amidoamine)-conjugated alginate hydrogels as a platform to direct the behavior of MSCs.

## Materials and methods

### Alginate scaffold fabrication

High molecular alginates extracted from the stipes of the brown algae *Lessonia nigrescens* (LN) and *Lessonia trabeculata* (LT) (Alginatec, Riedenheim, Germany) were used for scaffold fabrication. Both alginate types were dissolved separately as 0.65% (w/v%) solutions in isotonic, 0.9% sodium chloride solution (NaCl; B. Braun, Melsungen, Germany) and mixed afterwards in equal parts to adjust a defined M/G ratio. Unless otherwise stated, 2D alginate layers were applied as scaffolds fixed on round standard cell culture treated, polystyrene-based coverslips (PC; Thermanox™, 13 mm in diameter, Thermo Fisher Scientific, Dreieich, Germany) treated with poly-L-Lysine (Sigma-Aldrich, Taufkirchen, Germany) as 1:5 dilution [v/v%] in phosphate buffered saline (PBS; Gibco, Karlsruhe, Germany) for 30 min at 37 °C. Then, 120 µL of alginate solution (LN/LT 1:1, 0.65% (w/v %)) were placed on top of the treated, dried coverslips, distributed using a pipette tip and gelled at room temperature (RT) for 20 min using 800 µL of a crosslinking solution containing 20 mM barium chloride, 115 mM sodium chloride and 5 mM L-Histidine (all from Sigma-Aldrich, Taufkirchen, Germany). The final alginate layers (ALG) with a thickness of 1.4 mm were washed three times and stored at 4°C in NaCl until usage. In case of spherical scaffolds alginate was treated as described previously [18]. Briefly, the alginate solution (LN/LT 1:1, 0.65% (w/v %)) was dispersed into small droplets using a coaxial air stream and crosslinked for 20 min in the barium chloride gelation bath. Excessive gelating agents were removed by washing the spherical alginate scaffolds three times with NaCl.

### Alginate scaffold surface modification

The surface modification was carried out in a two-step process. First, the alginate’s carboxylic groups were activated by aqueous carbodiimide chemistry resulting in the conjugation of N-hydroxysuccinimide (NHS) as described previously [20]. Subsequently, obtained alginate-NHS ester (ANHS) were coupled with PAMAM dendrimers constituted of an ethylenediamine core as generation 3.0 (PAMAM G 3.0, Sigma-Aldrich, Taufkirchen, Germany) in various quantities (1.6, 3.2, 32, 324 and 3245 nmol PAMAM/cm^2^ surface area) for 24 h at RT. Matrigel-coated alginate surfaces (AMG) were obtained after incubating ANHS with 0.01 mg Matrigel/cm^2^ (Corning, New York, USA) suspended in DMEM/F12 for 24 h at 37 °C. Final modified alginate surfaces were washed three times with NaCl.

### Raman spectroscopy

Confocal Raman spectroscopy was used to analyze the amide bond formation between alginate and PAMAM. The Raman setup consists of a 532 nm continuous wave solid state laser (Compass 315 M, Coherent Inc., California, USA) as a light source and a microscope (Eclipse LV100, Nikon, Tokyo, Japan) connected to a spectrometer (Shamrock 303, Andor, Belfast, UK) through a multimode fiber (AFS50/125Y, Thorlabs, New Jersey, USA). Modified alginate layers were placed on a glass slide and dried for 24 h at RT. Raman spectra from the sample were measured using an 40 × air objective with a numerical aperture of 0.9 (S Fluor, Nikon, Tokyo, Japan) and a laser power of 50 mW.

### Zeta potential measurements

To analyze the synthesized surface charges spherical (modified) ALG (50 µm in diameter, total surface area: 10 cm^2^) suspended in PBS were evaluated at 25 °C in a capillary cell (Malvern Instruments, Worcestershire, England) using the Zetasizer Nano-ZS PN3702 (Malvern Instruments, Worcestershire, England). Changes in zeta potentials due to protein adsorption were measured after 24 h incubation of spherical (modified) ALG suspended in DMEM/F12 supplemented with 10% FCS at 37 °C and subsequently washed with PBS before analyzing.

### Wettability analysis using captive bubble technique

Contact angles were measured with an OCA 20 device including a conventional goniometer contact angle apparatus with the addition of a bracket for flat samples (SHC 20), a glass cuvette (GC 40), an upward curved dispensing needle (SNC 052/026), a digital image capture and data analysis software (SCA 20) (all from DataPhysics Instruments, Filderstadt, Germany) at RT. Using the captive bubble approach the contact angle is determined by placing an air bubble (3 µL) onto the hydrogel surface, which is surrounded with NaCl. A digital image was captured 3 s after placing of the air bubble and analyzed using the SCA 20 software. For analyzing the contact angles after protein adsorption the alginate scaffolds were incubated in DMEM/F12 supplemented with 10% FCS at 37 °C for 24 h and subsequently washed with NaCl.

### Protein adsorption assays

#### Ellman’s reagent

5,5′-dithio-*bis*-(2-nitrobenzoic acid) (DTNB, Sigma-Aldrich, Taufkirchen, Germany) was used to quantify the sulfhydryl groups of adsorbed proteins. The surfaces were incubated in DMEM/F12 supplemented with 10% FCS at 37 °C for 24 h and washed with PBS. After the addition of 0.1 mM DTNB solution and 2 min of incubation at RT, the absorbance of the test samples was measured at 412 nm with the Infinite® F200 microplate reader (Tecan, Maennedorf, Switzerland).

#### Protein folding study using ANS

To monitor the protein conformational changes 8-anilino-1-napthalenesulfonic acid (ANS, Sigma-Aldrich, Taufkirchen, Germany) was bound to the exposed hydrophobic regions of the protein. First, the surfaces were incubated in DMEM/F12 supplemented with 10% FCS at 37 °C for 24 h and washed with PBS. Second, 0.1 mM ANS solution was applied to the scaffolds for 15 min at RT. Third, the fluorescence of the test samples was determined at 470 nm with the Infinite® F200 microplate reader.

### Cell culture

Human mesenchymal stem cells (MSCs) (Wharton’s jelly, PromoCell GmbH, Heidelberg, Germany) were cultured and expanded in DMEM/F12 supplemented with 10% FCS, 100 units/mL penicillin/streptomycin and 1 ng/mL basic fibroblast growth factor (all from Gibco, Karlsruhe, Germany). Cells were passaged using 0.05% trypsin/EDTA (Gibco, Karlsruhe, Germany) once a week or at a confluency of ca. 80%. 2.5 × 10^5^ cells were seeded on (modified) alginate layers and cultivated for 24 h at 37 °C.

### Scanning electron microscopy (SEM), surface roughness and energy dispersive X-ray (EDX) microanalysis

#### SEM preparation

The samples before and/or after cell cultivation were washed in PBS, fixed at RT in a glutaraldehyde containing sodium cacodylate buffer (Carl Roth, Karlsruhe, Germany) and stored overnight at 4°C. SEM preparation was performed as described previously [29, 30]. Then, the samples were dehydrated in increasing alcohol concentration (from 10 to 100%) and dried in hexamethyldisilazane (HMDS, Sigma-Aldrich, Taufkirchen, Germany) as described previously [31]. Finally, all samples were coated with carbon, and studied in field emission scanning electron microscope Phillips FESEM XL30 (FEI, Eindhoven, Netherlands) at 5 kV in secondary electron (SE)-mode and at 10 kV accelerating voltage (10 mm working distances) in backscattered electron (BSE)-mode.

#### Determination of cell spreading

Cell spreading area was measured using the BSE-images according to the previously described automatic SEM-method [32]. Manual freehand selection and measuring/analyzing of cell area in ImageJ software were applied due to overlapping cells or minor contrast between cells and substrates [30]. Three substrates per condition were used and each experiment was repeated three times. More than 450 cells per condition were analyzed and the values were calculated considering HMDS-preparation shrinkage [31].

#### Surface roughness

The surface roughness was validated via an imaged-based quantification of surface characteristics and therefore classified by calculating the Haralick textural feature “entropy” (randomness of pixel distribution) [33]. Here, the roughness of the surface was abstracted by the image’s gray level heterogeneity. High entropy values correlated with rough surfaces (heterogeneous), whereas low entropy values correlated with smooth surfaces (homogeneous). The entropy of the BSE-images taken with the FESEM XL30 at 10 kV accelerating voltage and 10 mm working distance (119 × 121 px) was analyzed using the open source software Cellprofiler [34].

#### EDX-microanalysis

EDX-microanalysis was performed using the coupled FESEM software EDX Multi-Element Mapping (Version 3.35 from EDAX, Mahwah, New Jersey, USA). The measurements were carried out for 120 s, up to 20 times per condition.

### Cytotoxicity assay

MSCs grown for 24 h on polystyrene-based 96-well cell culture plates (Greiner Bio-One, Frickenhausen, Germany) were exposed to free and alginate-bound PAMAM for 24 h. Then, the CellTiter-Glo® luminescent cell viability assay normalized to the cell number of 9 × 10^4^ cells/ per condition was applied. The reagent was added in an equivalent volume to the amount of cell medium, mixed for 2 min and incubated for 10 min at RT. The emitted luminescent signal was detected using the Infinite® F200 microplate reader. The cytotoxicity of alginate-bound PAMAM was determined via a live/dead cell viability assay using fluorescein diacetate (FDA; Invitrogen, Karlsruhe, Germany) and ethidium bromide (EB; Invitrogen, Karlsruhe, Germany) imaged with the fluorescence microscope Nikon Eclipse TE300 (Nikon, Tokyo, Japan). To ensure an equal cell seeding on different alginate samples the cell suspension (2.5 × 10^5^ cells/ scaffold) was prepared by pre-dilution of the cells in the medium and was subsequently added to the alginate scaffolds after the removal of the scaffold-surrounding liquid/NaCl.

### Immunofluorescence staining

MSCs (2.5 × 10^5^ cells/ scaffold) grown on control and PAMAM-modified surfaces for 24 h were fixed with BD Cytofix™ fixation buffer (BD Biosciences, California, US) for 20 min at RT. After rinsing with PBS, the fixed cells were permeabilized with 0.2% TritonX (Sigma-Aldrich, Taufkirchen, Germany) in PBS for 20 min at RT. Then, the samples were rinsed with PBS and blocked with 1% bovine serum albumin (Sigma-Aldrich, Taufkirchen, Germany) and 0.2% TritonX in PBS for 30 min at RT. The immunofluorescence signals were gained after incubating for 24 h at 4 °C with Bodipy® FL phallacidin (Molecular Probes, Eugene, Oregon, USA) against F-actin, with anti-vinculin antibody (Abcam, Cambridge, UK) for vinculin staining and 20 min before observation with NucBlue® (Molecular Probes, Eugene, Oregon, USA) for nuclei staining. Images were taken using the confocal laser microscope Leica TCS SP8 equipped with a UV laser and Leica Application Suite X software (all Leica Microsystems, Mannheim, Germany).

### Quantitative polymerase chain reaction (qPCR)

qPCR was carried out 24 h after cell seeding (2.5 × 10^5^ cells/ scaffold) to evaluate the gene expression of CD105 (ENG), CD90 (THY1), CD73 (NT5E), RUNX2, SOX9, PPARG, vinculin (VCL), integrin α5 (ITGA5) and tensin 1 (TNS1) of MSCs grown on control and PAMAM-modified surfaces (n = 4). Total RNA was extracted using the RNeasy Micro Kit (Qiagen, Hilden, Germany) following the manufacturer’s instructions, and RNA concentrations were quantified with a NanoDrop 2000 spectrophotometer (Thermo Fisher Scientific, Dreieich, Germany). Complementary DNA (cDNA) was synthesized using a high-capacity cDNA reverse transcription kit (Applied Biosystems, Foster City, California, USA). qPCR was performed in 10 µL of reaction volumes containing 2.5 ng cDNA using the QuantStudio 7 Flex and QuantStudio Real-Time PCR software v1.1 (both Applied Biosystems, Foster, City, California, USA). Used assays are presented in the supplementary information (Supplementary Table 1). Gene expression was normalized to GAPDH and calculated using the comparative threshold cycle (Ct) method (relative gene expression = 2^(−ΔCt(sample) − ΔCt(control)^). Standard deviation of ΔΔCt values was calculated based on Gaussian error propagation (SD (ΔΔCt) = sqrt(SD ΔCt^2^(sample) + SD ΔCt^2^(control)). MSCs grown on polystyrene-based cell culture flasks (Cellstar®, Greiner Bio-One, Frickenhausen, Germany) were used as the control for the gene expression experiments.

### Statistical evaluation

Graphical illustration of data and statistical analyses were performed using OriginPro and IBM SPSS Statistics. Differences between groups were considered significant by p < 0.05 and were evaluated with univariate analyses of variance (ANOVA) with simple contrasts. In terms of cell spreading area analyses (Fig. 3b) an ANOVA with posthoc corrections was applied.

## Results

### Characterization of PAMAM conjugation to alginate hydrogels

**Fig. 1:**
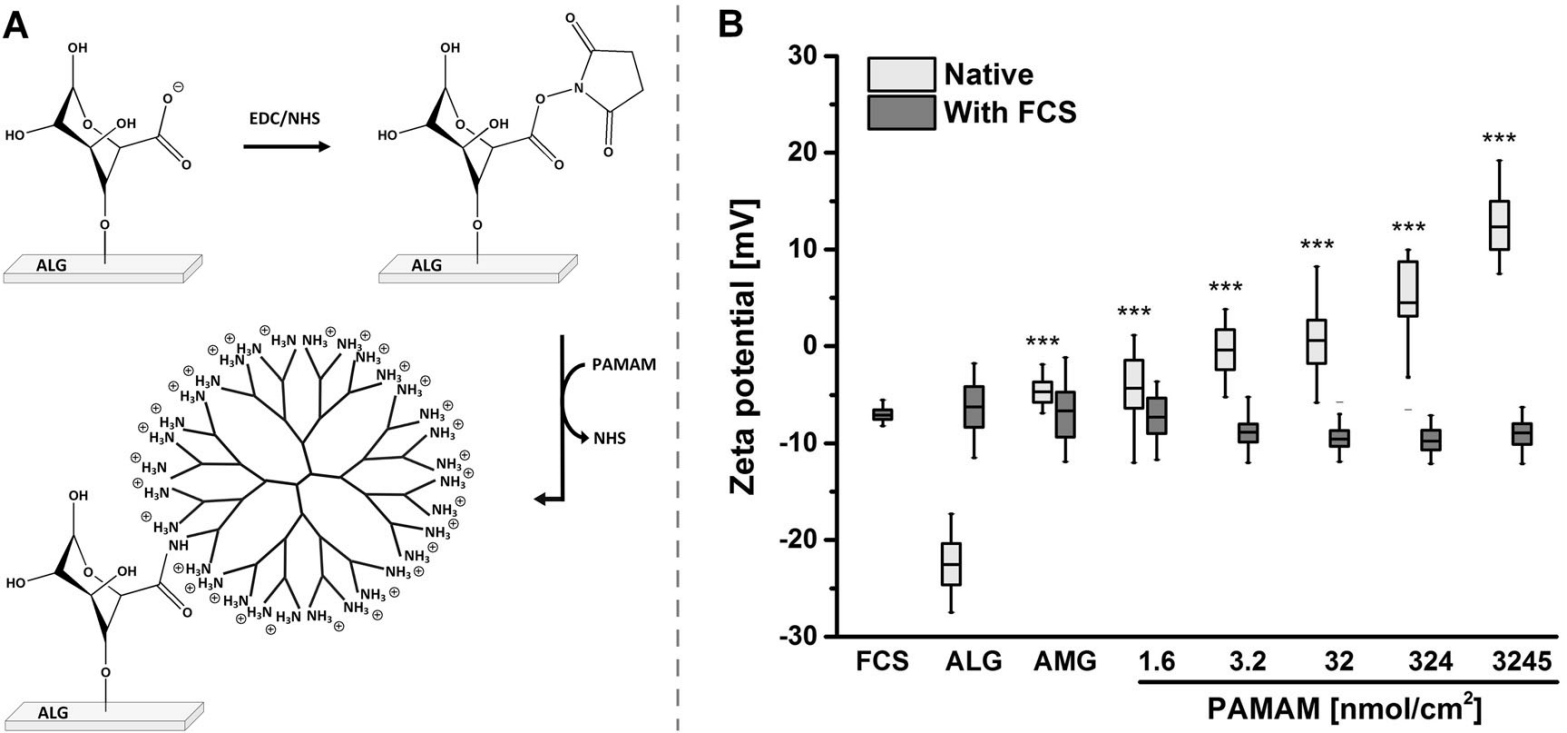
**a** Schematic illustration of the conjugation of poly(amidoamine) (PAMAM) to alginate hydrogel surfaces (ALG) via aqueous carbodiimide chemistry using 1-ethyl-3-(3-dimethylaminopropyl)carbodiimide (EDC) and N-hydroxysuccinimide (NHS). **b** Zeta potential analyses of fabricated native and fetal calf serum (FCS)-treated surfaces presented as box plots with mean line and percentiles. Native surfaces were statistically compared with ALG (***p < 0.001) (*n* ≥ 3)

Positively charged PAMAM were conjugated reproducibly to the surface of ALG via aqueous carbodiimide chemistry (Fig. 1a, Table 1). The scaffold modification was examined using energy dispersive X-ray (EDX) analyses displaying the increased nitrogen content at the alginate surface (Table 1). At high PAMAM concentrations (324 and 3245 nmol/cm^2^) the values for nitrogen saturated at 7.6 wt%. These results were supported by Raman spectroscopy data revealing the formation of amide bounds between alginate and PAMAM (Supplementary Fig. 3). The Raman signals spanning from 1555 cm^−1^ to 1742 cm^−1^ corresponded to the C = O stretching mode of the amide I bond. An integration of this band quantified the amount of linked PAMAM (Table 1). Moreover, analyses of the adsorptive behavior of PAMAM to alginate showed that PAMAM not only bound to alginate, but also form a multilayer at high concentrations (see Supplementary Fig. 1).

**Table 1:** Characterization of PAMAM coupling via EDX and Raman spectroscopy as well as surface analyses via contact angle measurements and entropy-based surface roughness analysis (mean ± SD, *n* ≥ 3)

**Sample**		**EDX**	**Raman**	**Contact angle**		**Surface roughness**	
		Nitrogen content (Wt%)	Peak area (amide I band) (a.u.)	Native (°)	With FCS (°)	Entropy	
						Native (unitless)	With FCS (unitless)
	ALG	1.82 ± 0.55	1.49 ± 0.15	45.77 ± 2.28	35.26 ± 4.59	2.95 ± 0.10	2.92 ± 0.12
	AMG	n.a.	n.a.	39.90 ± 5.72	33.21 ± 3.11	1.43 ± 0.19***	1.48 ± 0.30***
	PC	n.a.	n.a.	39.80 ± 0.95**	34.35 ± 0.50	1.84 ± 0.18***	1.98 ± 0.19***
PAMAM (nmol/cm^2^)	1.6	2.19 ± 0.78**	1.59 ± 0.66	34.19 ± 2.06***	35.80 ± 1.59	3.51 ± 0.09***	3.42 ± 0.16***
	3.2	2.32 ± 0.93**	2.65 ± 0.64	33.20 ± 3.02***	35.08 ± 1.55	2.91 ± 0.15	3.43 ± 0.10***
	32	3.42 ± 1.34	3.01 ± 0.84	34.46 ± 3.78***	36.92 ± 2.02	2.65 ± 0.09***	2.44 ± 0.11***
	324	7.57 ± 1.24***	6.22 ± 0.82***	36.25 ± 3.53**	36.62 ± 1.78	2.54 ± 0.32***	2.31 ± 0.12***
	3245	7.63 ± 1.16***	6.31 ± 2.01***	38.15 ± 7.51	35.92 ± 2.91	1.55 ± 0.22***	1.86 ± 0.17***

Differences of groups compared to ALG were considered significant by ^*^p < 0.05, ^**^p < 0.01 and^ ***^p < 0.001 (*n* ≥ 3)

*PAMAM* poly(amidoamine), *EDX* energy dispersive X-ray, *SD* standard deviation, *NHS* N-hydroxysuccinimide, *a.u.* arbitrary unit, *FCS* fetal calf serum, *ALG* alginate, *AMG* matrigel-coated alginate hydrogel, *PC* polystyrene-based coverslips, *n.a.* not available

### Surface charge analyses

Hydrogel surface charges were investigated using zeta potential measurements (Fig. 1b). Alginate gels bearing carboxylic groups exhibited a negatively charged surface (−22.5 ± 2.7 mV). The following amidation with positively charged PAMAM units resulted in increasing positive charges up to 12.2 ± 3.0 mV. After proteins attached to differently charged surfaces, the zeta potentials of all treated surfaces were equalized at −8.01 ± 2.38 mV (Fig. 1b). These results are consistent with the charge of unbound proteins (FCS, −7.09 ± 0.61 mV).

### Surface wettability and roughness

The captive bubble method was used to characterize the surface wettability (Table 1). The alginate hydrogel surface was found to be hydrophilic. The conjugation of PAMAM to ALG resulted in even more hydrophilic interfaces independently from the PAMAM concentration. After protein adsorption all surfaces exhibited a similar contact angle at 35.60 ± 2.85 °, which meets the wettability of amino group-bearing surfaces modified with PAMAM (Table 1).

The entropy of the BSE-images (Supplementary Fig. 2) was associated with the roughness of the imaged hydrogel surfaces (Table 1). The unmodified alginate hydrogel surface possessed about 70 nm small pores (Supplementary Fig. 2: *2A*/*B*). After the introduction of PAMAM, rough surfaces were observed at low concentrations (1.6 and 3.2 nmol PAMAM/cm^2^). However, the roughness of PAMAM-modified surface decreased with the increase of PAMAM-concentration (compare Supplementary Fig. 2, from *4A/B* to 8*A/B*, Table 1). Resulted smooth interfaces at high PAMAM concentrations (3245 nmol/cm^2^; 1.55 ± 0.22) were comparable with the analyzed entropy of standard cell culture treated, polystyrene-based coverslips (PC, 1.84 ± 0.18) and Matrigel-coated alginate surfaces (AMG; 1.43 ± 0.19). Protein adsorption did not affect the surface roughness as presented in Table 1 and Supplementary Fig. 2.

### Quantity and conformational changes of adsorbed proteins

**Fig. 2:**
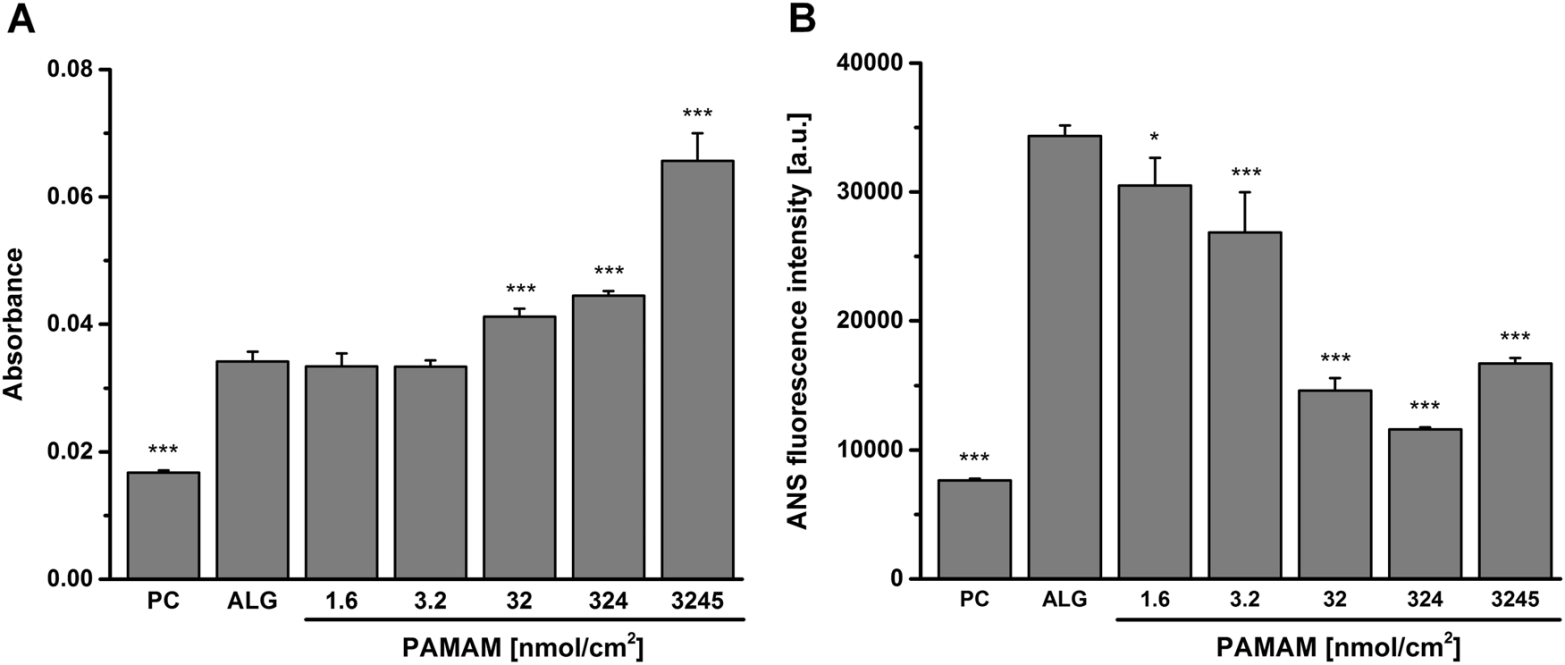
Protein adsorption studies revealed diverse protein scenarios on differently charged surfaces. **a** The surface-attached protein content was determined as the absorbance of the proteins sulfhydryl groups using Ellman’s reagent and grew with increasing PAMAM content. **b** Protein conformational changes were monitored with ANS conjugated to hydrophobic protein regions while emitting a fluorescence signal. High ANS fluorescence intensities were associated with a denaturation-related loss of proteins bioactive functionality. Differences of groups compared to ALG were considered significant by *p < 0.05 and ***p < 0.001 (*n* ≥ 3)

Ellman’s reagent was used to quantify the sulfhydryl groups of proteins adsorbed to native and modified alginate scaffolds incubated with serum-supplemented medium. As presented in Fig. 2a, protein adsorption occurred on all tested surfaces. While low amounts of proteins adsorbed on the cell culture plastic PC, increased protein adsorption was gained on alginate hydrogel surfaces. Low PAMAM concentrations (1.6 and 3.2 nmol/cm^2^) did not result in higher protein levels compared to pure ALG surfaces. However, enhanced protein adsorption was observed while increasing the amount of PAMAM (32, 324 and 3245 nmol/cm^2^).

The protein conformational changes were monitored using ANS, which bound to hydrophobic regions of proteins exposed due to folding/denaturation processes and displayed them via fluorescence emission. As presented in Fig. 2b, little protein conformational changes occurred to proteins adsorbed to PC. In contrast, adsorption to ALG surfaces resulted in high conformational changes. With increasing conjugation of PAMAM, however, the protein folding processes were reduced. Nevertheless, proteins adsorbed to high PAMAM concentrations (3245 nmol/cm^2^) altered their structure to a larger extent than 32 and 324 nmol/cm^2^ PAMAM.

### Cell spreading, morphology and surface features

**Fig. 3:**
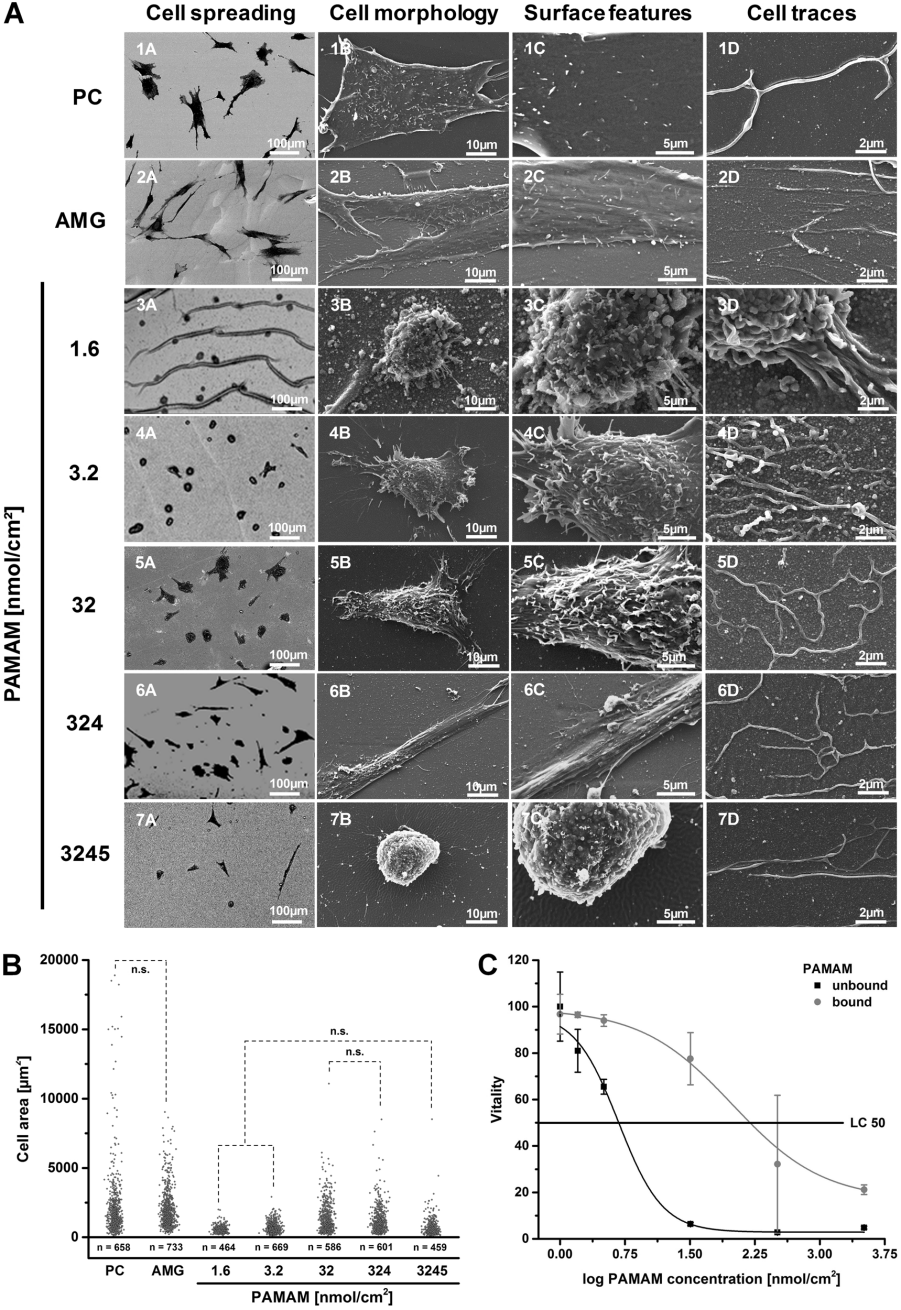
PAMAM-conjugated alginate surfaces were applied for MSC cultivation and were compared to standard cell culture treated, polystyrene-based coverslips (PC) and Matrigel-coated alginate surfaces (AMG) in terms of **a** cell spreading, cell morphology, surface features and cells traces as well as **b** cell spreading areas. Unless stated as not significant (n.s.) differences between groups were considered significant. **c** The cytotoxicity of unbound and bound PAMAM was found to be increased at high PAMAM concentrations and was attributed with the reduction of cell areas

**Fig. 4:**
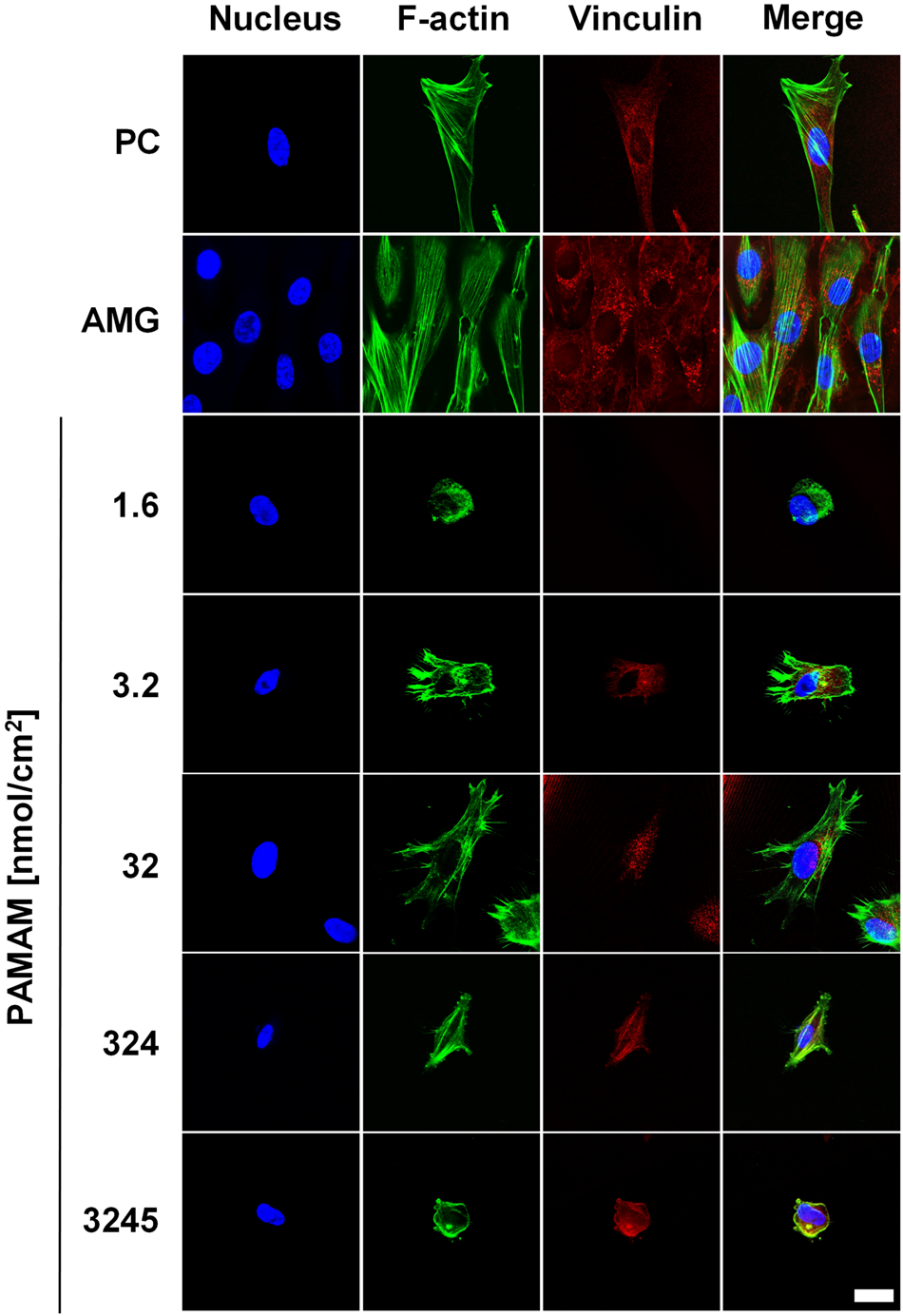
Representative images of the immunofluorescence staining against the cell nucleus, cytoskeleton (F-actin) and focal adhesion-related vinculin of fabricated non-PAMAM (PC, AMG) and positively charged PAMAM-surfaces. Nucleus volume and actin filaments shape correlated with the cell spreading area. Vinculin expression appeared to be independent from the materials surface charge. Scale bar represents 10 µm

PAMAM-conjugated alginate surfaces were examined in cell culture experiments with MSCs in comparison to PC and AMG (Figs 3 and 4). Cell spreading (Fig. 3a, *1A*–*7A*), cell morphology (Fig. 3a, *1B*–*7B*), cell surface features (Fig. 3a, *1C–7C*) as well as cell-substrate contacts and cell motility traces (Fig. 3a, *1D*–*7D*) were under examination. No cell adhesion occurred on ALG surfaces (Supplementary Fig. 5, 1A). Contrary, MSC attached to PC and AMG were well spread and exhibited a smooth cell surface relief covered with single, short microvilli (Fig. 3a, *1C/2C*). In both controls, long, thin tubes, which varied from 50 nm to 150 nm in diameter and showed numerous branches, were seen (Fig. 3a, *1D/2D*). In case of PAMAM-modified alginate surfaces, various cell behaviors linked to the PAMAM concentration were observed. On alginate surfaces conjugated with 1.6 nmol PAMAM/cm^2^ only round MSCs covered with a lot of microvilli, bubbles and ruffles were attached via very dense contacts and without visible cell traces (Fig. 3a, *3A–3D)*. Increasing the quantity of PAMAM up to 32 nmol/cm^2^ resulted in more star-shaped, spread cells with lengthened and branched cell traces (Fig. 3a, *4A–4D, 5A–5D*). Similar cell traces were observed at alginate surfaces modified with 324 nmol/cm^2^ PAMAM (Fig. 3a, *6D*), however, MSCs attached increasingly in a spindle-shaped form with a smoothened cell surface relief (Fig. 3a, *6A-6C*). A further increase of PAMAM concentration (3245 nmol/cm^2^) resulted in the rounding of attached MSCs with I) a smoothened surface relief bearing holes in the cell membrane, II) shortened and decreased microvilli, vesicles and wrinkles (Fig. 3a, *7A–7C*) and III) reduced cell motility traces (Fig. 3a, *7D*). Analyzing the area, with which the cells spread on differently PAMAM-modified alginate surfaces, a bell-formed trend with the highest area between 32 nmol/cm^2^ and 324 nmol/cm^2^ PAMAM concentration approximating to those of PC and AMG was displayed (Fig. 3b). It should be noted that some measured spreading areas have a great standard deviation due to cell division. Immunofluorescence staining against the cell nucleus, cytoskeleton (F-actin) and focal adhesion (vinculin) supports the observed bell-form trend (Fig. 4). The cell nuclei were small within round cells (1.6 and 3245 nmol/cm^2^ PAMAM) compared to larger ones of widely spread cells (32 nmol/cm^2^ PAMAM, PC, AMG). Furthermore, actin filaments were found to be short in round cells and lengthened with cell spreading area. Cytotoxicity assays revealed that highly positively charged PAMAM exhibited a cytotoxic effect on MSCs not only in an unbound state (LC50: 4.77 nmol/cm^2^), but also at high concentrations in surface-attached conditions (LC50: 156 nmol/cm^2^). Here, the cytotoxicity was substantially reduced due to PAMAM fixation to a surface (Fig. 3c).

### Gene expression analyses of adherent MSCs

**Fig. 5:**
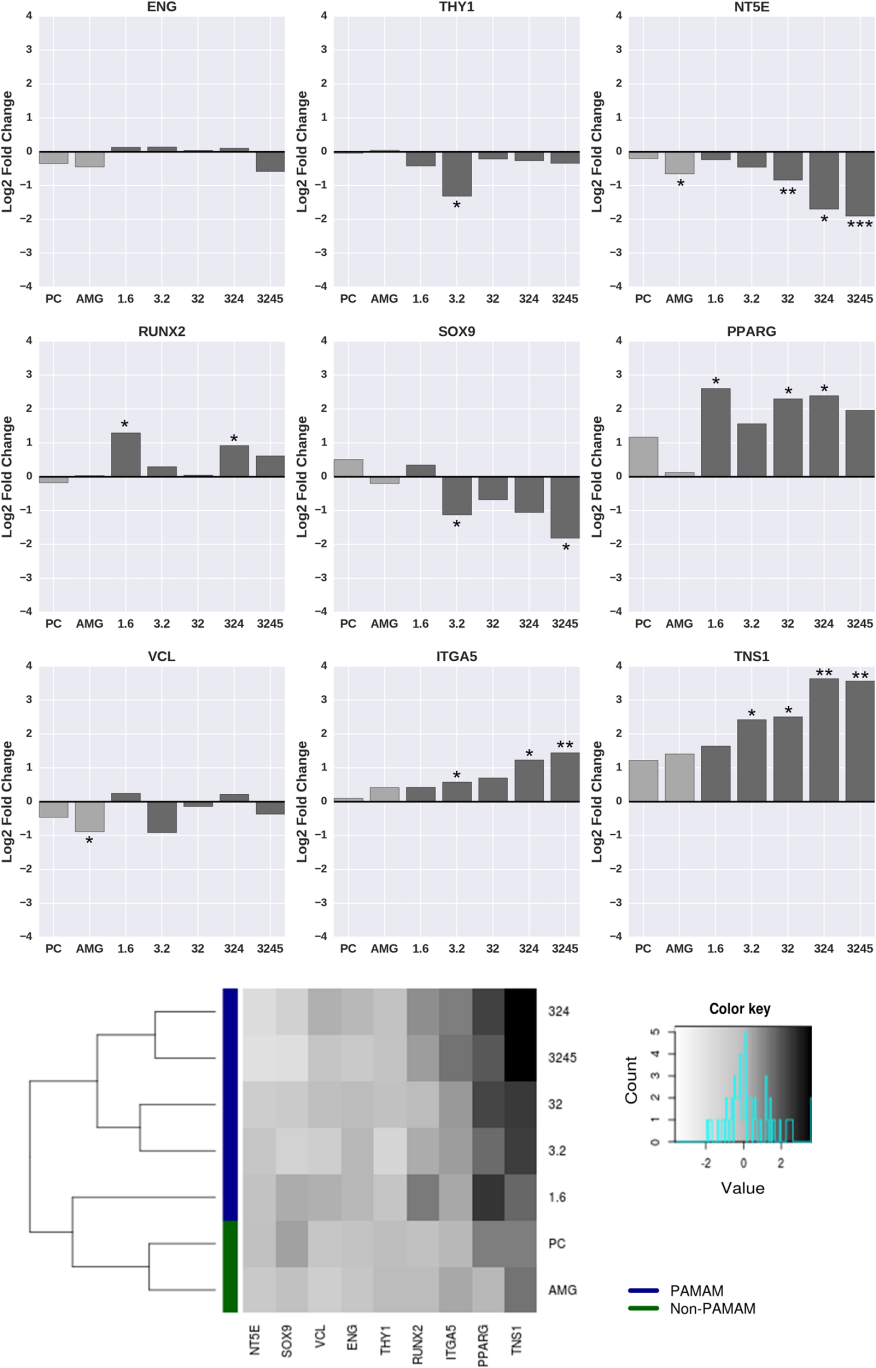
qPCR-resulted gene expression studies (*n* = 4) indicate the loss of MSCs phenotype (THY1 and NT5E downregulation) as well as the osteogenic (RUNX2) and adipogenic (PPARG) inducing effect of PAMAM while suppressing the chondrogenesis-associated gene SOX9. Adhesion-related genes integrin α5 and tensin 1 were found to be increased after adhesion. Differences of groups compared to the control were considered significant by *p < 0.05, **p < 0.01 and ***p < 0.001. Clustering of all gene expressions resulted in non-PAMAM and PAMAM groups hints at an impact of the surface charge on the adhesion, phenotype and differentiation of MSCs

In order to better understand the respective cell responses, gene expression analyses were performed via qPCR and compared to MSCs grown on polystyrene-based cell culture flasks as a control (Fig. 5). Here, the genes ENG, THY1 and NT5E were related to the phenotype of MSCs as proposed by the International Society for Cellular Therapy in 2006 [35]. The expression of ENG was similar for all conditions. However, the PAMAM-conjugation to ALG induced a downregulation of THY1 and NT5E. Besides MSC phenotype markers, the gene expression related to differentiation pathways were examined. RUNX2 as a gene expressed in the early stage of osteogenic induction (towards bone tissue) was found to be upregulated in MSCs grown on PAMAM-conjugated alginate surfaces. In contrast, chondrogenic differentiation (towards cartilage cells) analyzed using SOX9 expression was suppressed with increasing PAMAM content. In terms of adipogenic induction (towards fat tissue), MSCs grown on PAMAM surfaces showed an overexpression of the respective gene PPARG. Furthermore, adhesion-related gene expression of integrin α5 (ITGA5) and tensin 1 (TNS1) was enhanced with increasing PAMAM concentrations. Analyses concerning vinculin (VCL) revealed no clear change. Creating a heatmap of all qPCR data (Fig. 5) resulted in the clustering of the gene expressions into two groups: I) PAMAM and II) non-PAMAM excluding the lowest PAMAM-concentration (1.6 nmol/cm^2^). Here, similar clustering was displayed for I) the controls PC and AMG, II) 3.2 nmol/cm^2^ and 32 nmol/cm^2^ as well as III) 324 nmol/cm^2^ and 3245 nmol/cm^2^ PAMAM.

## Discussion

The surface charge of a biomaterial represents one of the key factors for the complex interaction of the engineered biomaterial with biological entities such as proteins and cells. To expand the insight of how surface charges impact the cellular behavior, we established a PAMAM-alginate hydrogel-based platform. Positively charged PAMAM was conjugated to the bioinert alginate hydrogel surface using carbodiimide chemistry. Here, the reproducible amidation between ALG and PAMAM was validated with the increase of PAMAM-attributed nitrogen content and the formation of amide bonds. Zeta potential measurements displayed that the introduced PAMAM molecules altered the surface charge from negatively charged alginate scaffolds to increasingly positive PAMAM surfaces. Here, each PAMAM G 3.0 molecule consists of 32 terminal amino-groups, which protonate in aqueous solutions to positively charged units. Thus, different surface charges spanning from -25 mV to + 15 mV were available for cell studies and expand the range of previously investigated charges [9, 10, 13, 14].

Moreover, protein adsorption was found to be tremendously affected by the surface charge. In aqueous solutions serum proteins were net negatively charged (-7.09 ± 0.61 mV, Fig. 1b) due to their low isoelectric point and possessed a hydrophilic periphery and a hydrophobic core [36]. The negative charge of protein units encouraged the adsorption to positively charged surfaces. Using Ellman’s reagent the sulfhydryl groups of adsorbed proteins were quantified and revealed an enhanced protein adsorption with increasing PAMAM-induced surface charge. Contrarily, proteins also attached on negatively charged ALG surfaces. Here, some positive protein domains may have interacted with ALG. Our ANS-based studies displayed a clear conformational change of ALG-adsorbed proteins. These alterations may be caused by the repulsive, electrostatic behavior of both negatively charged protein domains and ALG. The exposed positive protein domains could then interact with ALG leading to the adsorption of denatured proteins. In contrast, the PAMAM-induced increase of surface charge reduced the protein denaturation and hence supported the proteins bioavailability. We also demonstrated that the protein adsorption had an impact on the surface features. It should be highlighted that different surface charges and wettability were equalized after the protein coating. Hence, after protein adsorption all samples possessed negative zeta potentials similar to serum proteins and hydrophilic contact angles around 35.60 ± 2.85 °. Consequently, MSCs did not interact with surfaces possessing different characteristics like charge or wettability but rather with diverse protein compositions. However, the surface roughness appeared to be unaltered after protein adsorption.

The different protein conditions, which resulted due to specific surface charges, were applied for the cultivation of MSCs. Through direct binding to receptors within the cellular membrane MSCs interact with the surface-attached proteins. Even though proteins adsorbed to ALG surfaces, the material became bioinert due to substantial protein conformational changes. Excessive protein folding processes are associated with protein denaturation and result in a disturbed interaction with cells, since appropriate binding sites may be hindered. However, the PAMAM-associated increased protein content and reduction of protein conformational changes induced an enhanced cell attachment and spreading of MSCs with increasingly overexpression of integrin α5 and tensin 1. Here, the increase of potential binding sites for MSCs may be correlated with the cell spreading area, since both events show a related progress (compare Figs 2b and 3b). Nevertheless, cell spreading areas and vitality decreased at high PAMAM concentrations (324 and 3245 nmol/cm^2^). This phenomenon may be attributed to the cytotoxic effect of PAMAM, which potentially desorbed from the multilayer at high concentrations. This hypothesis is supported by the observed holes in the cell membrane and the reduction of cell traces at high PAMAM concentrations (3245 nmol/cm^2^), since cell traces are known to be left on the substrate due to cell migration and generally indicate the physiological and functional activity of cells [37]. Moreover, the decrease of the cell spreading at high PAMAM concentrations may also be correlated with the protein conformational changes (Figs 2b and 3b), since proteins adsorbed to high PAMAM concentrations (3245 nmol/cm^2^) altered their structure to a larger extent than 324 nmol/cm^2^ PAMAM and thus became less available for cell interactions.

Besides the adhesion behavior, our initial findings may indicate that the MSC’s fate was also affected by the hydrogels surface charge. Here, the suppression of the MSC-specific surface gene NT5E may display the loss of MSCs phenotype and the induction of differentiation processes as a result of increasing zeta potentials. The further analysis of RUNX2 as a marker for early osteogenesis hint that a positive surface charge may result in an osteogenic induction. These results are consistent with the present literature [7–10] and are in accordance with the upregulation of integrin α5, which is also attributed with osteogenesis of MSCs [38]. However, we further showed that the PAMAM-induced, increased surface charge may also have evoked the adipogenesis of exposed MSCs. Here, the adipogenic marker PPARG was found to be overexpressed in MSCs grown on PAMAM-conjugated alginate surfaces. Furthermore, the downregulation of SOX9 may indicate the suppression of the MSCs chondrogenic differentiation and is in accordance with the work of Wang and Sul, who claimed the downregulation of SOX9 as required for adipocyte differentiation [39]. All examined gene expressions clustered into two groups: I) PAMAM and II) non-PAMAM excluding the lowest PAMAM-concentration (1.6 nmol/cm^2^). Here, the quantity of 1.6 nmol/cm^2^ applied PAMAM was too low to result in a surface charge inducing a different gene expression compared to the controls. In contrast, the similarly clustered groups of 3.2/32 nmol/cm^2^ and 324/3245 nmol/cm^2^ PAMAM hint that a) the impact of the surface charge on the adhesion, phenotype and differentiation of MSCs and b) an increasing alteration of the respective cell behavior. Nevertheless, future gene expression studies with a wider array of genes and time points indicative of MSC differentiation are needed to draw final conclusions about how surface charge influences MSC differentiation. Here, functional assays, such as Alizarin Red staining and total calcium level (osteogenic potential), Safranin O staining (chondrogenic potential) and Oil Red O staining (adipogenic potential) are of particular interest.

To exclude that other surface features induced the cellular behavior, the surface wettability and roughness were investigated. Contact angle measurements revealed similar hydrophilic surfaces independently from the PAMAM concentration. Hence, an effect of wettability on the cell behavior was negligible. In contrast, the surface roughness associated with the entropy of the BSE-images decreased with increasing PAMAM content and has to take into account, since the surface roughness is known to effect MSC cultivation [40]. However, rough surfaces are also attributed with enhanced protein adsorption and cell adhesion [40] as well as the gene expression of RUNX2 and SOX9 downregulation [41], contrarily to the presented results in this study. Thus, the surface roughness appeared to be subordinated due to the dominant effect of the surface charge on protein adsorption and MSC behavior.

## Conclusion

Differently charged surfaces were fabricated by the conjugation of PAMAM to alginate-based hydrogels and enabled extensive studies regarding the effect of surface charges on protein adsorption and cell behavior of MSCs. Increasing surface charges resulted in enhanced quantities of biologically available, surface-attached proteins. Coating the biomaterial’s surface with proteins clearly showed an equalization of the surface wettability and charge. As a consequence, cells interacted rather with diverse protein compositions instead of different surface features. The different protein conditions were applied for the cultivation of MSCs and revealed an enhanced cell adhesion to increasingly positively charged surfaces. Gene expression studies may indicate the loss of MSC phenotype as well as the osteogenic and adipogenic inducing effect of positive surface charges while suppressing chondrogenesis-associated genes. The presented results affirm the important role of the biomaterials surface charge in terms of biointerface engineering and empower poly(amidoamine)-conjugated alginate hydrogels as a platform to direct the behavior of MSCs. These findings will benefit the development of biocompatible and functional materials, *e.g*. for the design of biomedical implants, in order to direct cellular behavior.

## Electronic supplementary material

Supplemental Information
