# Supplementary material for: Poly(amidoamine)-alginate hydrogels: directing the behavior of mesenchymal stem cells with charged hydrogel surfaces
Source: J Mater Sci Mater Med. 2018 Jun 30;29(7):105. doi: 10.1007/s10856-018-6113-x (PMC6028859; doi:10.1007/s10856-018-6113-x)
Supplement: Supplementary file 1 — Supplemental Information [file 10856_2018_6113_MOESM1_ESM.docx]

Supplementary Data for the following manuscript submitted to the

Journal of Materials Science: Materials in Medicine

Poly(amidoamine)-alginate hydrogels: Directing the behavior of mesenchymal stem cells with charged hydrogel surfaces

André Schulz ^a^, Alisa Katsen-Globa ^a^, Esther J. Huber ^a^, Sabine C. Mueller ^a^, Asger Kreiner ^a^, Norbert Pütz ^b^, Michael M. Gepp ^a^, Benjamin Fischer ^a^, Frank Stracke ^a^, Hagen von Briesen ^a^, Julia C. Neubauer ^a^, Heiko Zimmermann ^a,c,d^

*Affiliation*

^a^ Fraunhofer Institute for Biomedical Engineering, Joseph-von-Fraunhofer-Weg 1, Sulzbach 66280, Germany

^b^ Faculty of Medicine, Saarland University, Kirrberger Straße 100, Homburg 66421, Germany

^c^ Chair for Molecular and Cellular Biotechnology, Saarland University, Saarbruecken 66123, Germany

^d^ Faculty of Marine Science, Universidad Católica del Norte, Coquimbo, Chile

*Corresponding author*

Prof. Dr. Heiko Zimmermann,

Joseph-von-Fraunhofer-Weg 1, Sulzbach 66280, Germany

[heiko.zimmermann@ibmt.fraunhofer.de](mailto:heiko.zimmermann@ibmt.fraunhofer.de)

Phone: +49 6897 9071 100

Fax: +49 6897 9071 110

**Supplementary Table 1.** Assay IDs of the applied qPCR probes.

| **Gene** | **Assay ID** |
| --- | --- |
|  |  |
| GAPDH | Hs99999905_m1 |
| ENG | HS00923996_m1 |
| THY1 | Hs00264235_s1 |
| NT5E | Hs00159686_m1 |
| RUNX2 | Hs01047973_m1 |
| SOX9 | Hs01001343_g1 |
| PPARG | Hs00234592_m1 |
| VCL | [Hs00419715_m1](https://www.thermofisher.com/taqman-gene-expression/product/Hs00419715_m1?CID=&ICID=&subtype=) |
| ITGA5 | [Hs01547673_m1](https://www.thermofisher.com/taqman-gene-expression/product/Hs01547673_m1?CID=&ICID=&subtype=) |
| TNS1 | [Hs00917032_m1](https://www.thermofisher.com/taqman-gene-expression/product/Hs00917032_m1?CID=&ICID=&subtype=) |

## Adsorption isotherm

To study the adsorptive behavior of PAMAM to alginate surfaces, ALG incubated in PAMAM solutions with different concentrations (100, 200, 324, 648 and 1296 nmol/cm^2^ alginate surface) for 24 h at RT. Then, the absorbance of the supernatant was measured at 280 nm and RT using an UV/VIS spectrometer (Fullerton, California, USA) and disposable UV-cuvettes (1.5 mL, semi-micro, Dimensions: 12.5 x 12.5 x 45 mm; Brand, Wertheim, Germany) to determine the amount of unbound PAMAM and hence to calculate the quantity of adsorbed PAMAM. The plot of adsorbed PAMAM against the supernatant PAMAM resulted into the adsorption isotherm.


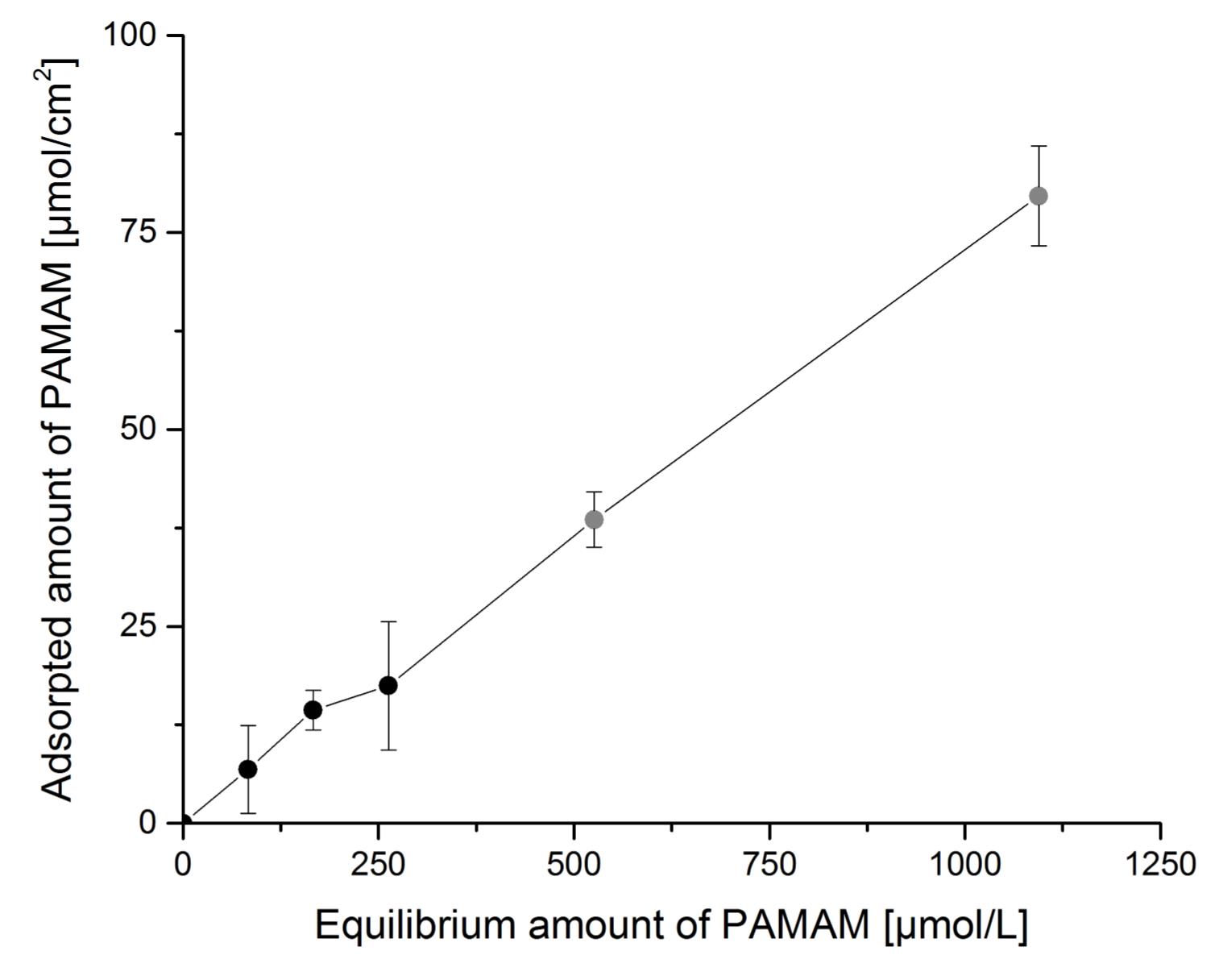


**Supplementary Fig. 1.** Adsorption behavior of PAMAM to ALG. The progression of the graph is characteristic for a multilayer formation based on the Brunauer, Emmett & Teller (BET) theory. PAMAM adsorbed increasingly until saturation of the surface (monolayer; black dots). After the saturation of available binding sites the PAMAM adsorption extended to the formation of a multilayer at high PAMAM concentrations (grey dots).


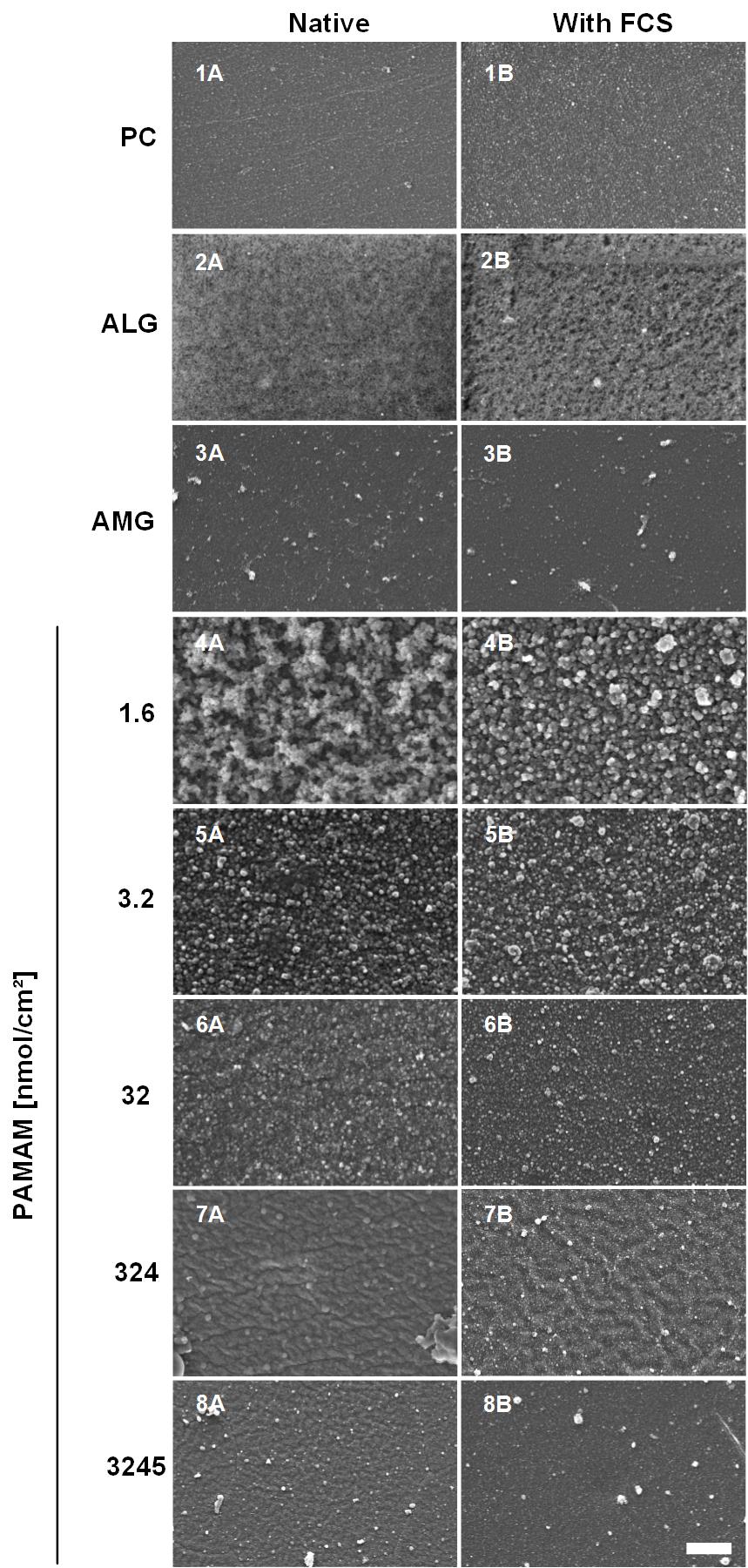


**Supplementary Fig. 2.** Representative BSE-images of fabricated native and FCS-treated surfaces displaying a decreased surface roughness with increasing PAMAM-conjugation. Scale bar represents 2 µm.


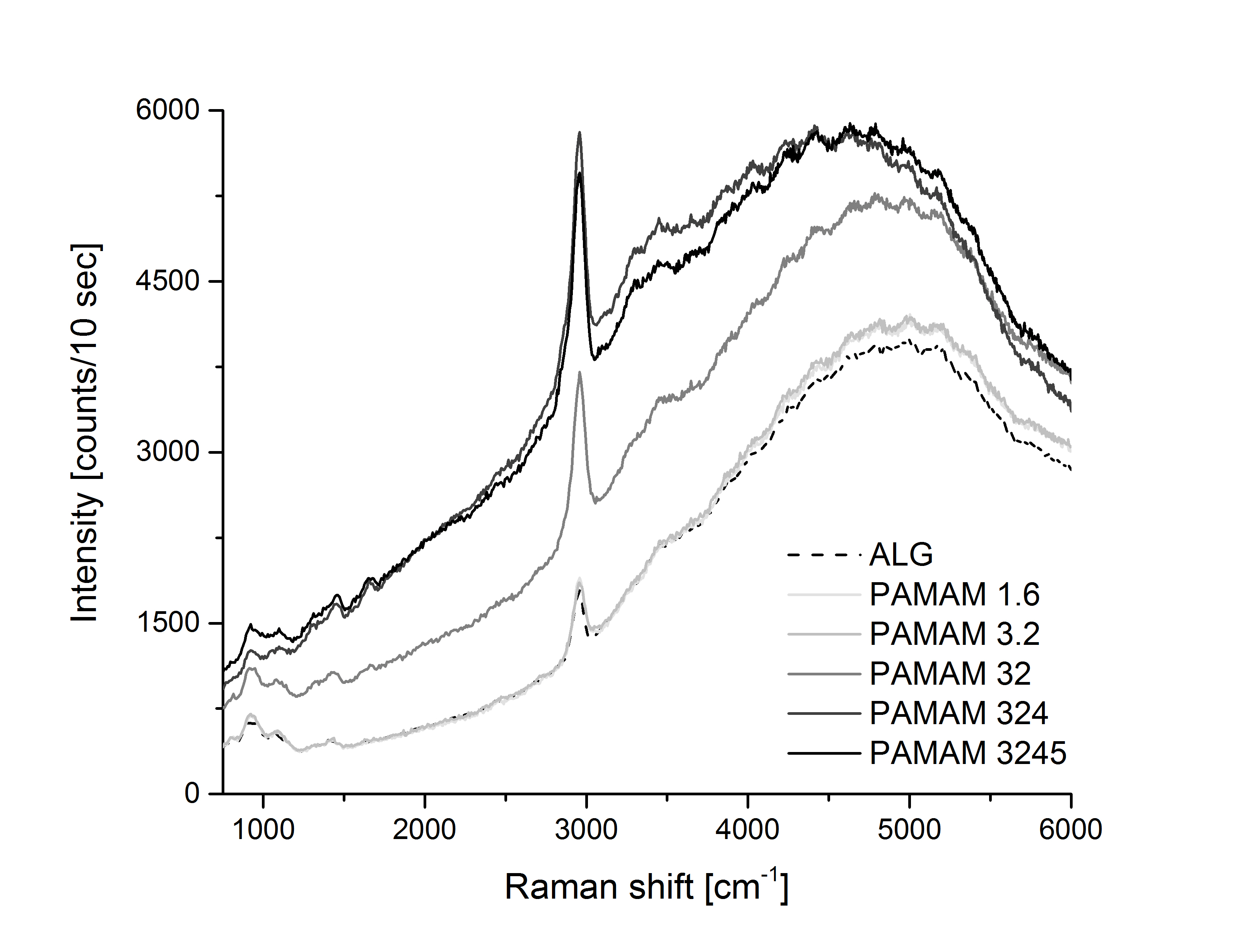
**Supplementary Fig. 3.** Representative Raman spectra from unmodified (ALG) and PAMAM-conjugated alginates.


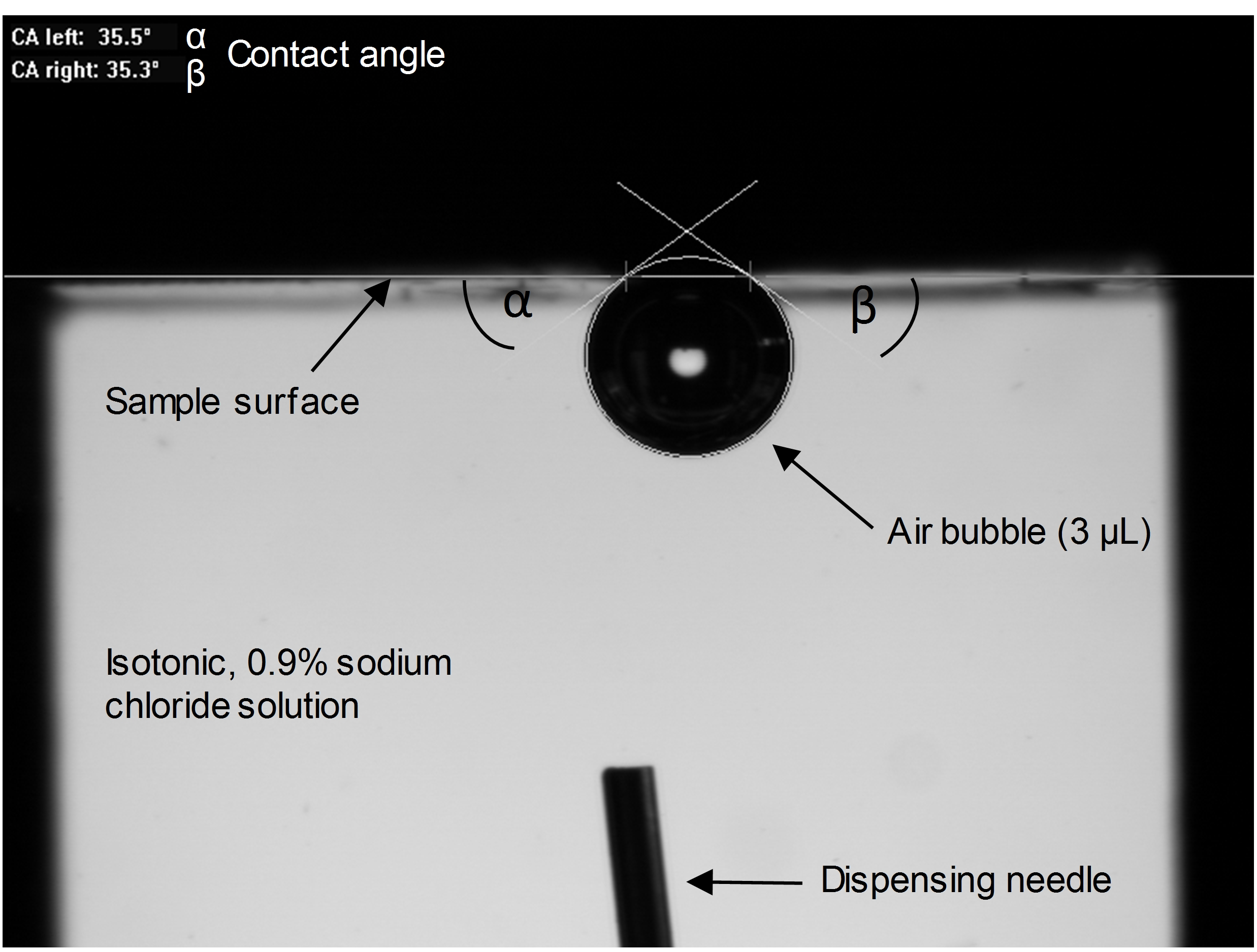


**Supplementary Fig. 4.** Representative image of the contact angle setup. Using the captive bubble technique the surface wettability was analyzed by placing an air bubble onto the hydrogel surface surrounded by isotonic sodium chloride solution.


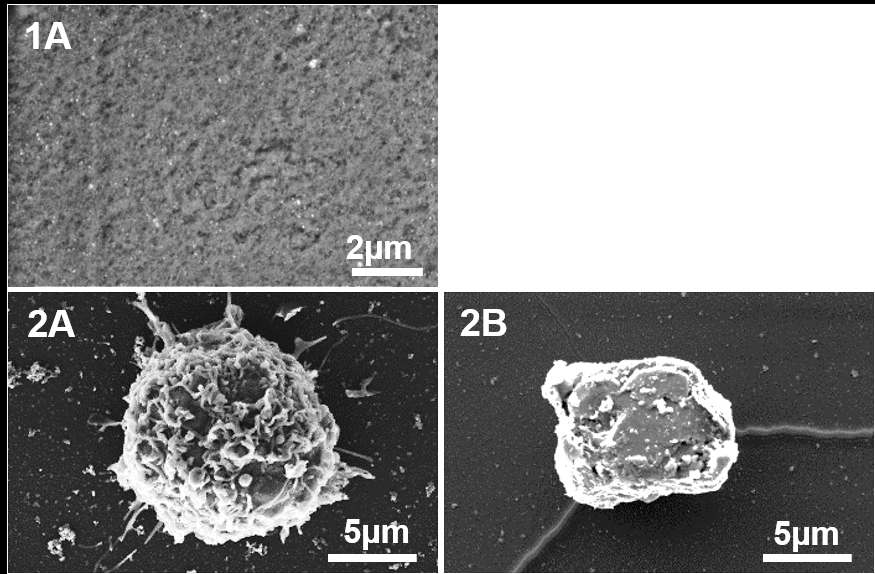


**Supplementary Fig. 5.** Representative SEM images of MSCs 24 h after seeding. No cell adhesion occurred on bioinert alginate surfaces (1A). On NHS-linked alginate (2A, 2B) some round cells with disturbed surfaces covered with microvilli, ruffles and vesicles were found. The formless cells with numerous holes in the plasma membrane indicated the cytotoxicity of EDC/NHS.

**Supplementary Table 2.** Standard deviations (SD) of ΔΔCt values of the qPCR studies (n =4).

| **SD** | **ENG** | **THY1** | **NT5E** | **RUNX2** | **SOX9** | **PPARG** | **VCL** | **ITGA5** | **TNS1** |
| --- | --- | --- | --- | --- | --- | --- | --- | --- | --- |
| **PC** | 0.37 | 0.25 | 0.33 | 0.30 | 0.46 | 0.80 | 0.31 | 0.31 | 0.39 |
| **AMG** | 0.40 | 0.51 | 0.30 | 0.31 | 0.38 | 0.82 | 0.29 | 0.24 | 0.42 |
| **1.6** | 0.71 | 0.69 | 0.73 | 0.54 | 0.73 | 0.73 | 0.48 | 0.37 | 0.41 |
| **3.2** | 0.33 | 0.35 | 0.25 | 0.30 | 0.37 | 0.47 | 0.5 | 0.26 | 0.43 |
| **32** | 0.29 | 0.38 | 0.34 | 0.35 | 0.51 | 0.72 | 0.31 | 0.42 | 0.44 |
| **324** | 0.72 | 0.61 | 0.61 | 0.53 | 0.68 | 0.82 | 0.64 | 0.53 | 0.44 |
| **3245** | 0.37 | 0.41 | 0.44 | 0.44 | 0.66 | 0.82 | 0.37 | 0.43 | 0.39 |
